# Left ventricular assist device implantation and clinical outcomes in the Netherlands

**DOI:** 10.1007/s12471-023-01760-9

**Published:** 2023-02-01

**Authors:** Kevin Damman, Kadir Caliskan, Ozcan Birim, Michiel Kuijpers, Luuk C. Otterspoor, Aria Yazdanbakhsh, Meindert Palmen, Faiz Z. Ramjankhan, Lauren F. Tops, Linda W. van Laake, Working Group on Mechanical Circulatory Support of the Netherlands Society of Cardiology and the Netherlands Society of Cardiothoracic Surgeons

**Affiliations:** 1 University of Groningen, Department of Cardiology, University Medical Centre Groningen, Groningen, The Netherlands; 2 Department of Cardiology, Erasmus University Medical Centre, Rotterdam, The Netherlands; 3 University of Groningen, Department of Cardiothoracic Surgery, University Medical Centre Groningen, Groningen, The Netherlands; 4 Heart Centre, Catharina Hospital, Eindhoven, The Netherlands; 5 OLVG, Amsterdam, The Netherlands; 6 Department of Cardiothoracic Surgery, Leiden University Medical Centre, Leiden, The Netherlands; 7 Amsterdam University Medical Centre, Amsterdam, The Netherlands; 8 Department of Cardiothoracic Surgery, University Medical Centre Utrecht, Utrecht, The Netherlands; 9 Department of Cardiology, Leiden University Medical Centre, Leiden, The Netherlands; 10 Department of Cardiology, University Medical Centre Utrecht, Utrecht, The Netherlands

**Keywords:** Heart failure, Left ventricular assist device, European Registry for Patients with Mechanical Circulatory Support, Heart transplantation

## Abstract

**Background:**

Left ventricular assist device (LVAD) therapy is an established treatment for advanced heart failure with reduced ejection fraction. We evaluated the characteristics and clinical outcomes of patients implanted with an LVAD in the Netherlands.

**Methods:**

Patients implanted with an LVAD in the Netherlands between 2016 and 2020 were included in the analysis. Baseline characteristics entered into this registry, as well as clinical outcomes (death on device, heart transplantation) and major adverse events (device dysfunction, major bleeding, major infection and cerebrovascular event), were evaluated.

**Results:**

A total of 430 patients were implanted with an LVAD; mean age was 55 ± 13 years and 27% were female. The initial device strategy was bridge to transplant (BTT) in 50%, destination therapy (DT) in 29% and bridge to decision (BTD) in the remaining 21%. After a follow-up of 17 months, 97 (23%) patients had died during active LVAD support. Survival was 83% at 1 year, 76% at 2 years and 54% at 5 years. Patients implanted with an LVAD as a BTT had better outcomes compared with DT at all time points (1 year 86% vs 72%, 2 years 83% vs 59% and 5 years 58% vs 33%). Major adverse events were frequently observed, most often major infection, major bleeding and cerebrovascular events (0.84, 0.33 and 0.09 per patient-year at risk, respectively) and were similar across device strategies. Patients supported with HeartMate 3 had a lower incidence of major adverse events.

**Conclusions:**

Long-term survival on durable LVAD support in the Netherlands is over 50% after 5 years. Major adverse events, especially infection and bleeding, are still frequently observed, but decreasing with the contemporary use of HeartMate 3 LVAD.

**Supplementary Information:**

The online version of this article (10.1007/s12471-023-01760-9) contains supplementary material, which is available to authorized users.

## What’s new?

- Left ventricular assist device (LVAD) implantation is increasingly being used in the Netherlands as therapy for advanced, end-stage heart failure.
- LVAD outcomes in the Netherlands are similar to findings from international registries.
- Overall, survival after LVAD implantation is good, but the incidence of adverse events remains high.

## Introduction

Heart failure with reduced ejection fraction (HFrEF) is a syndrome associated with poor quality of life, signs and symptoms of congestion, frequent admissions to the hospital and a high mortality risk. Typically, HFrEF is managed by evidence-based pharmacotherapy and device therapy when necessary [1]. About 1–10% of patients with HF (in general) can be labelled as “advanced” HF [2]. In selected patients, advanced treatment options such as heart transplantation and mechanical circulatory support (MCS) by a left ventricular assist device (LVAD) may be an option [1, 3, 4]. Specifically in the Netherlands, a relatively large donor heart shortage results in a long average time on the waiting list for heart transplantation, increasing the duration of MCS support, even in transplant candidates [5]. LVAD therapy has therefore become an important strategy to bridge patients with advanced HF as safely as possible to transplantation or to improve prognosis and quality of life in destination therapy (DT) patients [4, 6]. In the current study we investigated the characteristics and clinical outcomes of patients implanted with a durable LVAD in the Netherlands between 2016 and 2021.

## Methods

In the Netherlands LVAD therapy may be considered in patients who are either (future) candidates for heart transplantation or DT, as outlined in the 2019 consensus document [4]. The implantation of an LVAD, including the device brand, is entirely at the discretion of the treating physician. It is mandatory in the Netherlands to include all patients in the European Registry for Patients with Mechanical Circulatory Support (EUROMACS) after their signed informed consent has been obtained [4, 7]. For this analysis we included all patients in the Netherlands that were implanted with a durable LVAD in the period from 1 January 2016 to 31 December 2020 and were registered in EUROMACS. Patients underwent LVAD implantation at four centres: Erasmus University Medical Centre Rotterdam, Leiden University Medical Centre (LUMC), University Medical Centre Groningen and University Medical Centre Utrecht. LUMC only implanted LVADs as DT, whereas the other three centres implanted the device as a bridge to transplant (BTT), bridge to decision (BTD) or as DT. All patients provided informed consent for inclusion in EUROMACS and associated research as described above.

### Primary outcomes

We evaluated time to all-cause mortality during active LVAD support as a primary outcome variable. As secondary outcome measures we evaluated time to heart transplantation and the occurrence of specific major adverse events as defined by EUROMACS criteria as (1) device dysfunction, including LVAD pump thrombosis, (2) major bleeding, (3) major infections (including device-related infections), and cerebrovascular accidents. For these adverse events we evaluated time to first events and the total number of events (including repeating events) per person-time.

### Statistical analyses

Normally distributed continuous variables are presented as mean ± standard deviation, non-normally distributed variables as median and 25th–75th percentile. Categorical variables are presented as numbers (percentage) [*n* (%)]. Differences in baseline characteristics based on device strategy were evaluated using either *t*-test, chi-square or Mann-Whitney U test, where appropriate. Outcomes for time to event analysis were visually depicted by Kaplan-Meier curves. Differences between groups in survival analysis were evaluated using Cox regression analysis. Incidence rates of major adverse events were depicted as event rates per patient-year at risk. Comparison for incidence rates was obtained by chi-square test using the stir command in STATA. Two tailed *p*-values < 0.05 were considered statistically significant. Statistical analyses were performed using STATA SE 14.2 (STATA Corp., College Station, TX, USA).

## Results

**Fig. 1:**
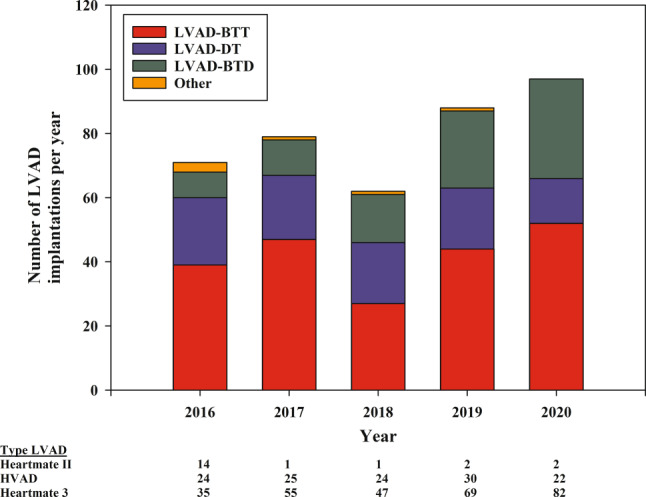
Number of left ventricular assist device (*LVAD*) implantations in the Netherlands according to device strategy in the last 5 years. *BTT* bridge to transplant, *DT* destination therapy, *BTD* bridge to decision

From 2016 to 2020, a total of 430 patients received a durable LVAD in the Netherlands, of whom 27% were female and the mean age was 55 ± 13 years. The number of LVAD implantations increased from 73 in 2016 to 106 in 2020 (Fig. 1). BTT was the most frequent device strategy at implantation (50%), while BTD and DT were less frequent (21% and 29%, respectively). Tab. 1 shows the baseline characteristics of the study population, stratified by initial device strategy.

**Table 1:** Baseline characteristics of the study population according to device strategy

	Overall	BTT	BTD	DT	*p*-value
*n*	430	215	89	126	
Age (years)	55 ± 13	51 ± 13	52 ± 12	64 ± 7	< 0.001
Female/male (%)	27/73	29/81	35/65	20/80	0.043
Ethnicity (Caucasian %)	95	92	95	97	0.48
BMI (kg/m^2^)	26 ± 5	25 ± 5	27 ± 5	26 ± 4	0.004
LVEF (%)					0.60
– < 20	73	79	75	68	
– 20–30	21	15	24	23	
– > 30	5	5	2	8	
– Unknown	1	1	0	2	
TAPSE (mm)	16 ± 4	17 ± 4	17 ± 4	15 ± 3	0.12
Aetiology of heart failure (%)					< 0.001
– Ischaemic	40	14	37	55	
– DCM	51	76	58	35	
– HCM	3	10	0	1	
– Valvular	1	0	2	2	
– Congenital	2	0	0	5	
– Other	2	0	3	2	
INTERMACS level (%)					< 0.001
– 1—Critical cardiogenic shock	9	8	11	1	
– 2—Sliding fast	28	33	34	14	
– 3—Inotrope dependent	34	33	29	41	
– 4—Resting symptoms	24	24	18	30	
– 5—Housebound	5	2	5	11	
– 6—Walking wounded	0	0	0	0	
– 7—Advanced NYHA III	0	0	3	0	
Type LVAD (%)					< 0.001
– HeartMate II	5	7	1	4	
– HVAD	24	26	14	46	
– HeartMate 3	71	67	85	50	
Temporary MCS before LVAD (%)	25	24	37	12	0.005
Medical history (%)					
– Diabetes mellitus	18	11	22	28	< 0.001
– COPD	6	2	4	12	0.24
– Cancer other than local skin cancer	8	2	8	14	0.087
Medical therapy^a^ (%)					
– Inotropes before implant	94	99	91	90	< 0.001
– ACEi/ARB	32	48	22	30	0.013
– Beta-blocker	54	62	45	58	0.17
– MRA	70	76	66	68	0.47
– Loop diuretic	93	90	91	97	0.33
– Amiodarone	46	44	48	46	0.92
– ICD	73	72	63	84	< 0.005
Length of stay after implantation (days)	27 (21–38)	25 (20–34)	30 (21–44)	28 (23–42)	0.002

*ACEi* angiotensin-converting enzyme inhibitors, *ARB* angiotensin II receptor blocker, *BMI* body mass index, *BTD* bridge to decision, *BTT* bridge to transplant, *COPD* chronic obstructive pulmonary disease, *DCM* dilated cardiomyopathy, *DT* destination therapy, *HCM* hypertrophic cardiomyopathy, *HVAD* HeartWare ventricular assist device,* ICD* implantable cardioverter defibrillator, *INTERMACS* Interagency Registry for Mechanically Assisted Circulatory Support, *LVAD* left ventricular assist device, *LVEF* left ventricular ejection fraction, *MCS* mechanical circulatory support, *MRA* mineralocorticoid receptor antagonist, *NYHA* New York Heart Association class of heart failure, *TAPSE* tricuspid annular plane systolic excursion

^a^Data from two hospitals only

Patients who were implanted with an LVAD as a BTT were younger, more often female, had a lower body mass index and higher Interagency Registry for Mechanically Assisted Circulatory Support (INTERMACS) levels. Their aetiology was dilated cardiomyopathy in the majority of cases, while they also had fewer comorbidities such as pulmonary disease, diabetes or a history of cancer. Patients who were implanted with an LVAD as DT had a mean age of 64 ± 7 years, with a range of 37–75 years. Patients with an LVAD as a BTD had the highest frequency of temporary MCS before permanent LVAD implantation (37%).

The most frequently implanted LVAD was the HeartMate 3(Abbott) (66%), followed by the HeartWare ventricular assist device (HVAD; Medtronic) (29%), while the number of HeartMate II (Abbott) implantations is decreasing fast (5% in the past 5 years). Whereas HeartMate 3 is the most frequently used device as a BTT, the HVAD has been implanted more often as a DT strategy. The reason is that one of the four implanting centres in the Netherlands is a dedicated DT centre which only implanted the HVAD and was responsible for 42% of DT implantations in the Netherlands in the last 5 years.

### Clinical outcome with LVAD therapy

**Fig. 2:**
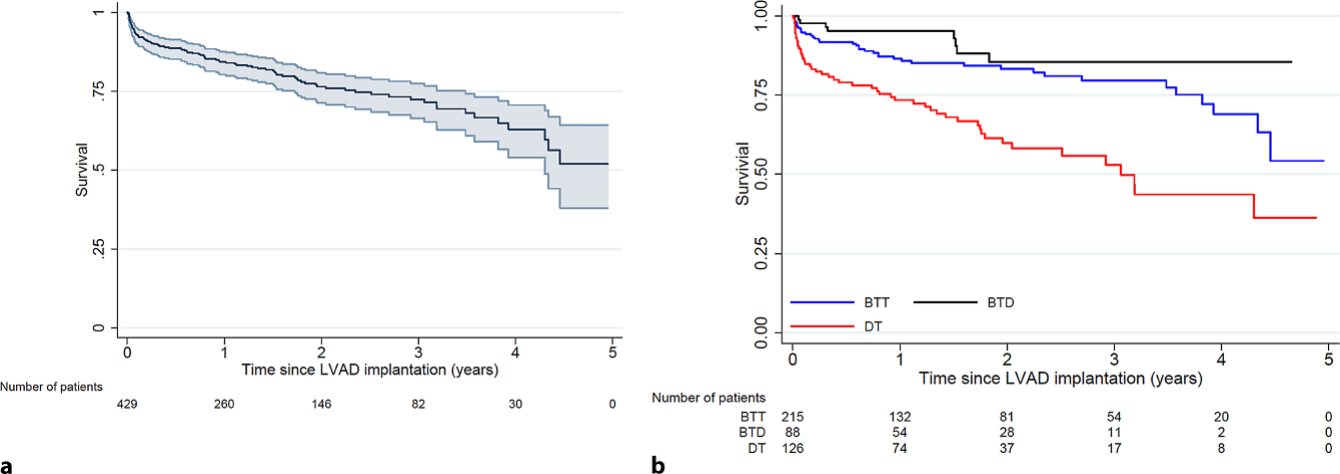
**a**, **b** Overall survival on active device support. **a** Overall survival on left ventricular assist device (*LVAD*) support. **b** Overall survival on LVAD support stratified by device strategy. Hazard ratio for bridge to transplant (*BTT*) vs bridge to decision (*BTD*) 0.55 (0.26–1.18), *p* = 0.13; BTT vs destination therapy (*DT*) 2.44 (1.61–3.72), *p* < 0.001; DT vs BTD: 0.40 (0.27–0.62), *p* < 0.001

After a median follow-up period of 17 months (maximum 59.4 months), 97 (23%) patients had died during active LVAD support. Survival status was not available for 1 patient. For the overall cohort, on-device survival was 83 (79–87)% at 1 year, 76 (71–80)% at 2 years, 73 (67–78)% at 3 years and 54 (41–66)% at 5 years, respectively (Fig. 2a). The on-device survival for the different device strategies (BTT, BTD, DT) is depicted in Fig. 2b, with a significantly better survival for BTT and BTD compared with DT. The 1‑, 2‑ and 5‑year on-device survival probabilities were 86 (80–90)%, 83 (76–88)% and 58 (38–74)% for an LVAD as a BTT, 95 (86–98)%, 86 (73–93)% and 86 (73–93)% for an LVAD as a BTD, and 72 (63–79)%, 59 (49–68)% and 33 (15–53)% for an LVAD as a DT strategy, respectively.

In the same period, 41 patients received a heart transplant. Figure S1 (Electronic Supplementary Material) shows the competing risk of death on device therapy, heart transplantation or being alive on LVAD therapy for the subgroup of patients with an initial strategy of implanting an LVAD as a BTT.

### Major adverse events—time to first event

**Fig. 3:**
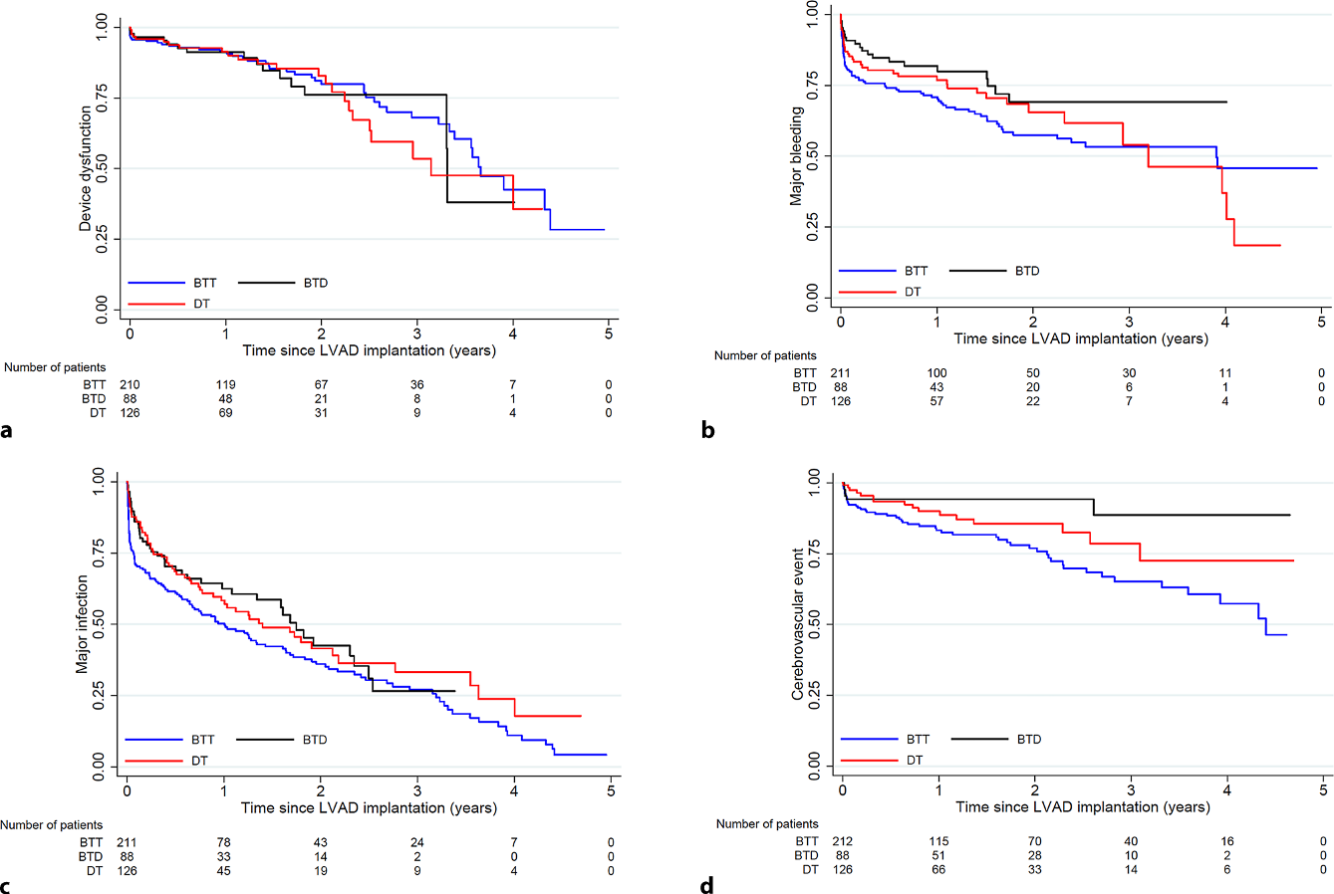
**a–d** Time to first major event. **a** Device dysfunction. Hazard ratio for bridge to transplant (*BTT*) vs bridge to decision (*BTD*) 1.06 (0.59–1.93), *p* = 0.83; BTT vs destination therapy (*DT*) 1.12 (0.68–1.86), *p* = 0.65; DT vs BTD: 0.89 (0.54–1.47), *p* = 0.65. **b** Major bleeding. Hazard ratio for BTT vs BTD 0.60 (0.37–0.98), *p* = 0.042; BTT vs DT 0.85 (0.58–1.26), *p* = 0.43; DT vs BTD: 1.17 (0.79–1.72), *p* = 0.43. **c** Major infection. Hazard ratio for BTT vs BTD 0.73 (0.52–1.04), *p* = 0.08; BTT vs DT 0.73 (0.54–0.98), *p* = 0.04; DT vs BTD: 1.37 (1.01–1.86), *p* = 0.04. **d** Cerebrovascular events. Hazard ratio for BTT vs BTD 0.29 (0.12–0.68), *p* = 0.004; BTT vs DT 0.57 (0.33–1.00), *p* = 0.051; DT vs BTD: 1.74 (1.00–3.07), *p* = 0.051

Figure 3a–d depicts time to first major adverse event. The incidence of device dysfunction, including pump thrombosis, was similar across device strategies, and was around 25% after 2 years. Figure S2 (Electronic Supplementary Material) shows time to first event stratified by LVAD type, showing an increased risk of device dysfunction with both the HeartMate II and the HVAD as compared with the HeartMate 3. The cumulative incidence of time to first major bleeding event was 40% at 24 months, increasing to 62% after 5 years (Fig. 3b). This was mainly attributable to 15% bleeding events in the first 30 postoperative days. Crude data showed that patients with an LVAD as a BTT had the highest bleeding risk, but after adjusting for device brand, there was no significant difference between BTT and BTD/DT [adjusted hazard ratio (HR) 0.67 (0.40–1.10), *p* = 0.11 and HR 0.84 (0.57–1.25), *p* = 0.40, respectively]. Figure 3c shows the incidence of major infections, which includes device-related infections, and shows an incidence of 55% of major infections after 24 months. After adjustment for LVAD brand, patients with an LVAD as DT remained at lower risk of overall infection [HR 0.72 (0.53–0.97), *p* = 0.03]. Finally, Fig. 3d shows the incidence of cerebrovascular events, which overall was 17% after 24 months. Patients with an LVAD as a BTT were at the highest risk of cerebrovascular events, but this was entirely attributable to the use of the HVAD in this group of patients (Fig. S3a, b, Electronic Supplementary Material). Figure S4 (Electronic Supplementary Material) shows the cumulative incidence and time to first event of any major adverse event (bleeding, infection, cerebrovascular events and device dysfunction). Freedom from any major adverse event was 42% at 1 year, 27% at 2 years and 9% at 5 years.

### Major adverse events—incidence of repeated events

Table 2 and Table S1 (Electronic Supplementary Material) show the cumulative incidence per person-year for the major adverse events during follow-up. The most frequent adverse event was major infection, occurring in 0.74 per patient-year at risk, and accounting for over 600 events in total during follow-up. In this case, major infection accounts for all infections, not specific device-related infections. Major bleeding was the second most frequent (cumulative) major event, followed by device dysfunction and cerebrovascular events. All events were more frequently observed with the HVAD/HeartMate II as compared with the HeartMate 3.

**Table 2:** Major adverse events on left ventricular assist device (*LVAD*) support stratified by device strategy

	Total	BTT	BTD	DT
*Major adverse event, n (per patient-year at risk)*				
– Device dysfunction/LVAD pump thrombosis	139 (0.15)	82 (0.16)	18 (0.12)	39 (0.16)
– Major bleeding	253 (0.33)	127 (0.31)	47 (0.33)	79 (0.38)
– Major infection	606 (0.86)	398 (0.95)*	117 (1.00)*	91 (0.49)
– Cerebrovascular events	85 (0.09)	63 (0.13)	8 (0.05)**	15 (0.06)**

*BTT* bridge to transplant, *BTD* bridge to decision, *DT* destination therapy

*p < 0.001 vs DT, **p < 0.05 vs BTT

## Discussion

In this study, evaluating outcomes of durable LVAD support in the Netherlands, we found that long-term survival was good and similar to international standards. Known major adverse events were frequently observed, with major infections and major bleeding occurring most often.

Advanced HF accounts for a small proportion of patients with HFrEF, but given the high prevalence of HF, the number of patients experiencing or developing advanced HF is large and increasing [2]. Typically, in outpatients with stable chronic HFrEF (New York Heart Association class I–IIIb), oral pharmacotherapy in combination with (biventricular) implantable cardioverter defibrillators, valves or coronary interventions are used to improve mortality and morbidity [1]. For those patients that develop advanced HFrEF LVAD therapy is often the only viable short-term option.

There are multiple possible reasons for the increase in the number of LVAD implantations in the Netherlands. First, since the end of 2015, LVAD as DT has been designated as standard insured care, increasing the number of patients implanted with an LVAD as DT [1, 4, 6]. Secondly, the number of heart transplantations in the Netherlands has been consistently low over the past years, whereas the heart transplant waiting list is growing fast [3, 5]. For many patients, this waiting time is too long to survive in a good clinical condition without MCS therapy. Finally, some patients are implanted with an LVAD as a BTD and eventually may become transplant candidates. This increase in the number of LVAD implantations is in contrast to recent developments in the United States, where an update in the heart transplant allocation system has resulted in a decrease of patients with an LVAD on the transplant waiting list (from 47% to only 14%) [8]. This is solely explainable by the much higher number of available donor hearts and heart transplants per capita in the United States. Although there are new opportunities for heart transplantation in the Netherlands (including donation after circulatory death heart transplantation and new donor registration legislation), these changes were only introduced in 2021, and the effects on LVAD implantations as a BTD have to be awaited [5].

Against this background, the survival of patients implanted with an LVAD in the Netherlands, even in those in whom a BTD strategy was implemented, was good in relation to the severity of the underlying condition. Compared with the published literature, survival in our cohort was similar to that in the INTERMACS registry for all device strategies [9]. In the MOMENTUM-3 study, evaluating HeartMate II versus HeartMate 3, the overall 24-month all-cause mortality was 79% in the HeartMate 3 group and 76% in the HeartMate II group, similar to the findings in the Netherlands [10]. Five-year survival in our cohort was slightly better at 54% compared with 43% in the INTERMACS registry.

LVAD therapy improves long-term survival and quality of life, but this comes at the cost of an increase in (device-related) major adverse events. Data from INTERMACS show that after 36 months over 85% of patients will experience a major adverse event. Most prevalent major adverse events are major (including gastro-intestinal) bleeding and major infection. Compared with data from INTERMACS, the rate of major bleeding was exactly the same (0.33 events per patient-year), while in our cohort the rate of major infection was higher (0.74 vs. 0.44 events per patient-year). Specifically, the number of patients experiencing a major infection directly after LVAD implantation was large in our study. These are often not device-related infections, but are associated with the complexity of medical management after LVAD implantation. Whereas bleeding remains an important issue considering the persisting need for therapeutic anticoagulation, the number of patients experiencing device dysfunction/pump thrombosis was significantly lower with the HeartMate 3. This device has now replaced the HeartMate II, as it was shown to be superior in the MOMENTUM‑3 trial based on the incidence of pump thrombosis and pump replacement [9]. Similarly, patients who were implanted with the HVAD experienced device dysfunction/pump thrombosis and cerebrovascular events more often. Whereas the HeartMate II has an axial flow pump design, which has shown to be susceptible to pump thrombosis, the HVAD is a centrifugal flow pump, with similarities to the HeartMate 3. However, the gaps where blood passes the rotor of the pump are smaller with the HVAD as compared with the HeartMate 3, possibly causing more haemolysis and acquired von Willebrand disease. Furthermore, in contrast to the HeartMate 3 the HVAD does not possess intrinsic pulsatility. These reasons may be the cause of the higher incidence of pump thrombosis and cerebrovascular events observed with this device. As a result, and following the observation that some patients experience severe malfunction of the HVAD, this device has not been commercially available since 2021. Therefore, the number of patients implanted with the HeartMate 3 is expected to increase further, which may result in a further decrease in overall device dysfunction over time [11]. This may also lead to a reduction in the number of major neurological events, as with both the HeartMate II and the HVAD the time to first and cumulative cerebrovascular event rates was significantly higher than with the HeartMate 3 [10, 12]. Overall, the burden of major adverse events in patients implanted with a durable MCS remains high.

### Limitations

This is a retrospective analysis of data from a European registry of durable LVAD implantations. The registration of patients in this registry is mandatory in the Netherlands. Differences between device strategy and types of LVAD should be seen as hypothesis generating, as there was no randomised allocation of devices, nor did we perform extensive correction or propensity score matching for pre-operative variables. Detailed information on the type of major events, including location of bleeding, infection or type of device dysfunction, was often not captured in the dataset. This could mean that the incidence of device-related events was overestimated. In EUROMACS, survival information is only captured while the patient is on LVAD support, and not thereafter. Overall survival, i.e. after transplantation, is therefore not available from these data.

## Conclusion

Long-term survival on durable LVAD support in the Netherlands is over 50% after 5 years and similar to international standards. Morbidity as indicated by major infection and bleeding are the most frequent occurring adverse events. Compared with other LVAD types, lower rates of device dysfunction and cerebrovascular events occur in patients implanted with the HeartMate 3.

## Supplementary Information

Supplementary Figures 1–4

Table S1. Major Adverse Events on LVAD support stratified by type of device
