# Supplementary material for: Left ventricular assist device implantation and clinical outcomes in the Netherlands
Source: Neth Heart J. 2023 Feb 1;31(5):189–95. doi: 10.1007/s12471-023-01760-9 (PMC10140239; doi:10.1007/s12471-023-01760-9)
Supplement: Supplementary file 1 — Supplementary Figures 1–4 [file 12471_2023_1760_MOESM1_ESM.docx]

**Supplementary Figure 1. Competing risk of death or heart transplantation in LVAD-BTT**

**
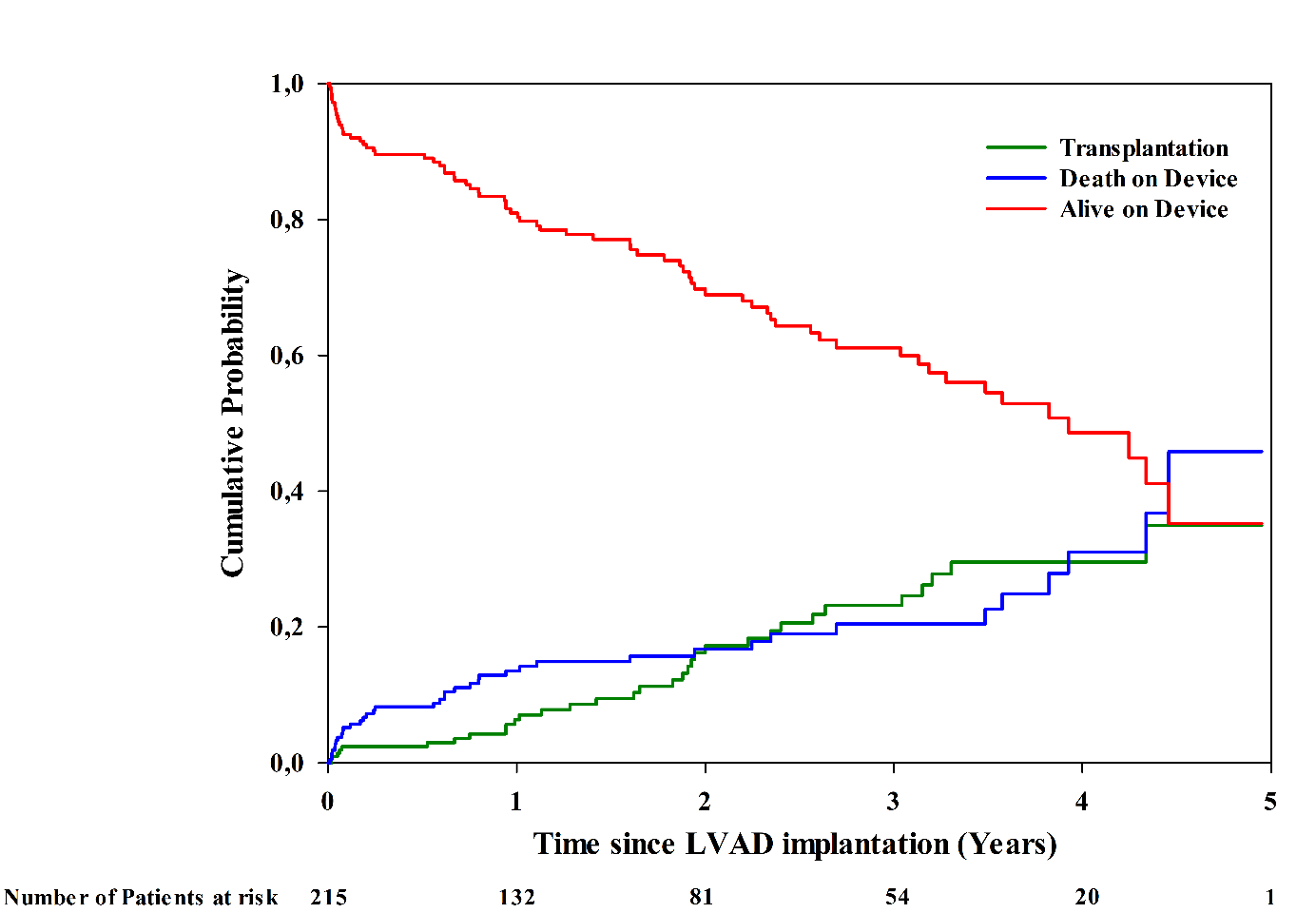
**

**Supplementary Figure 2 – Device dysfunction stratified by LVAD type**


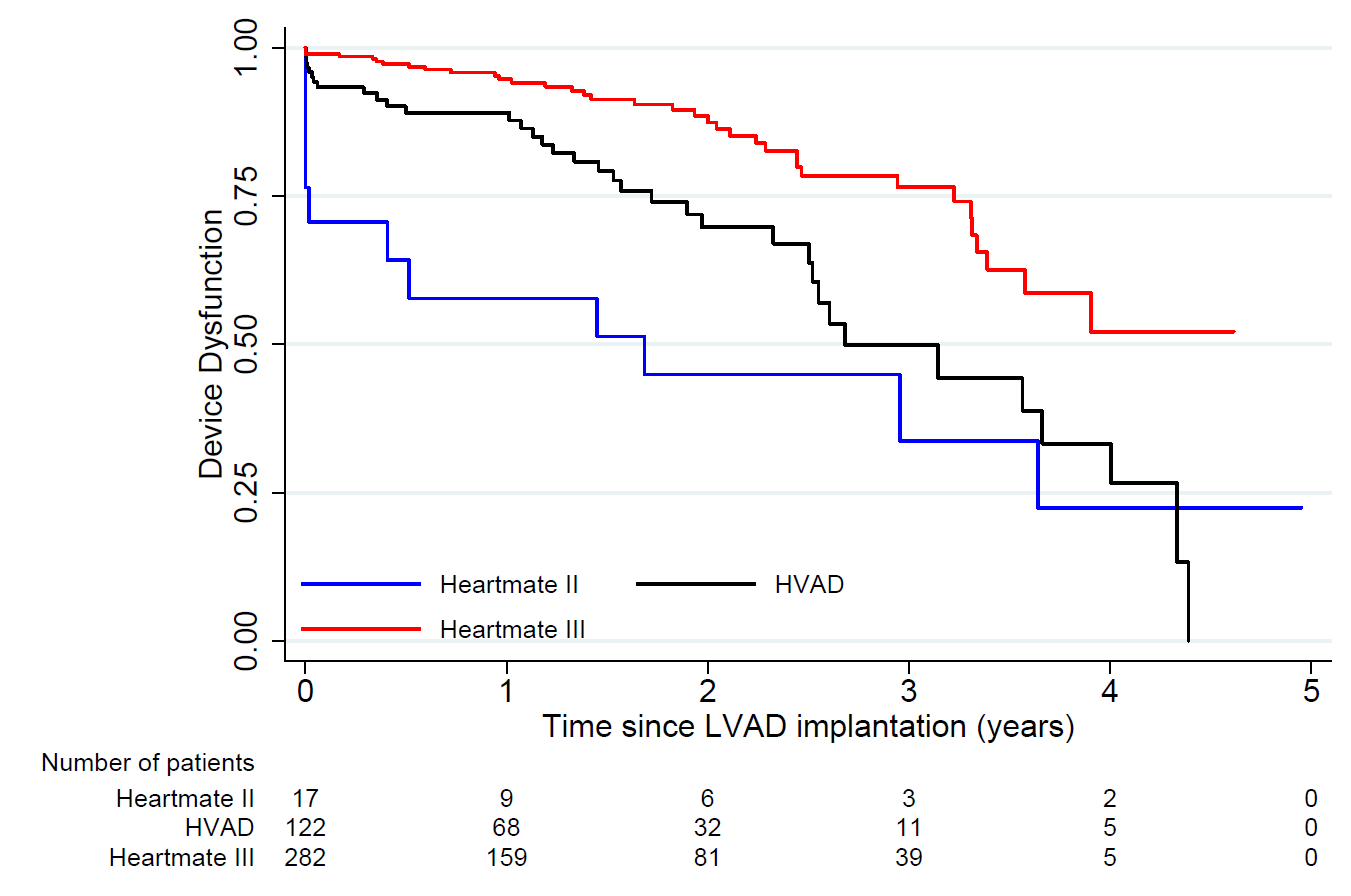


**Supplementary Figure 3A – Cerebrovascular events stratified by LVAD type (overall)**


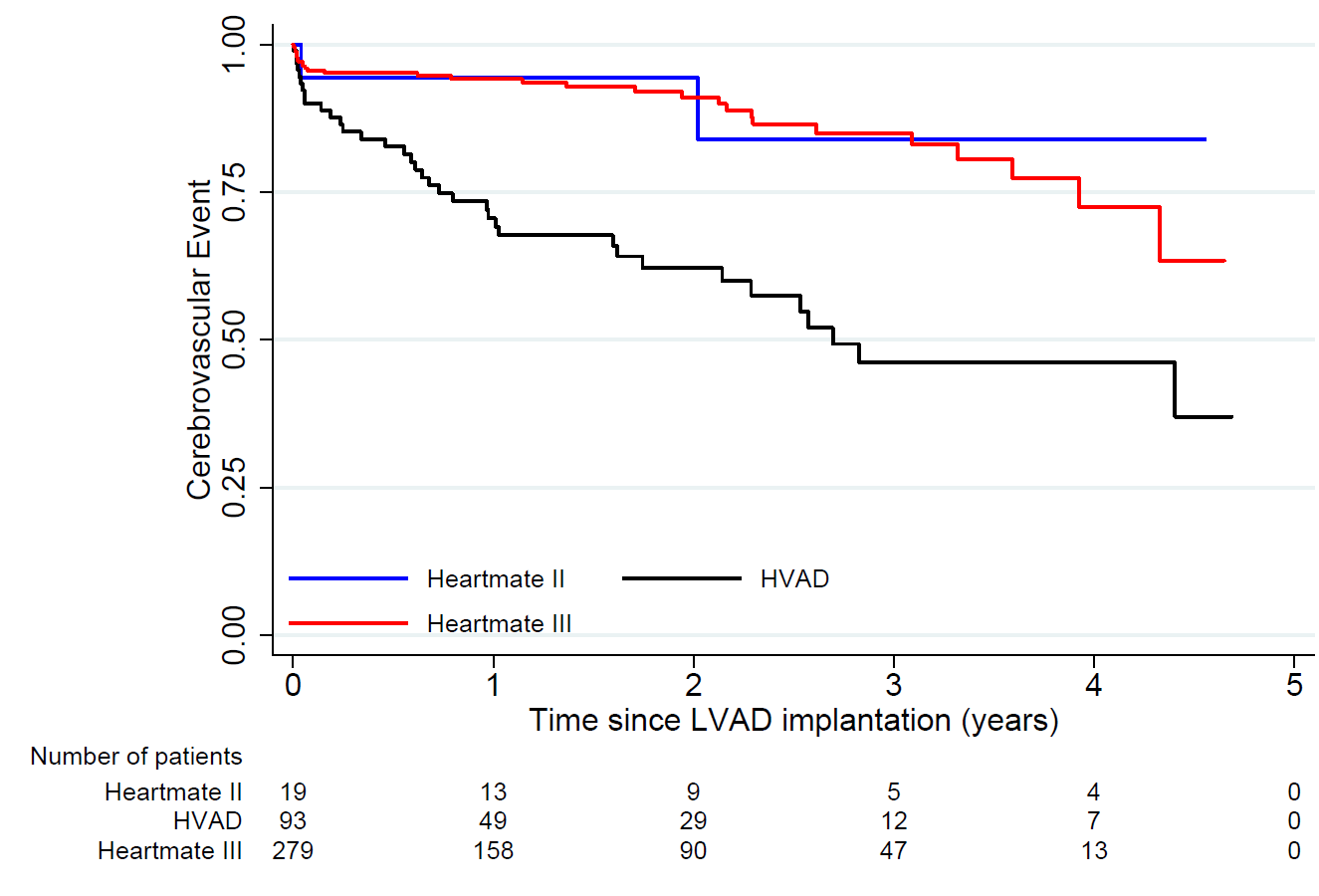


**Supplementary Figure 3B – Cerebrovascular events stratified by LVAD type in BTT patients only**

**
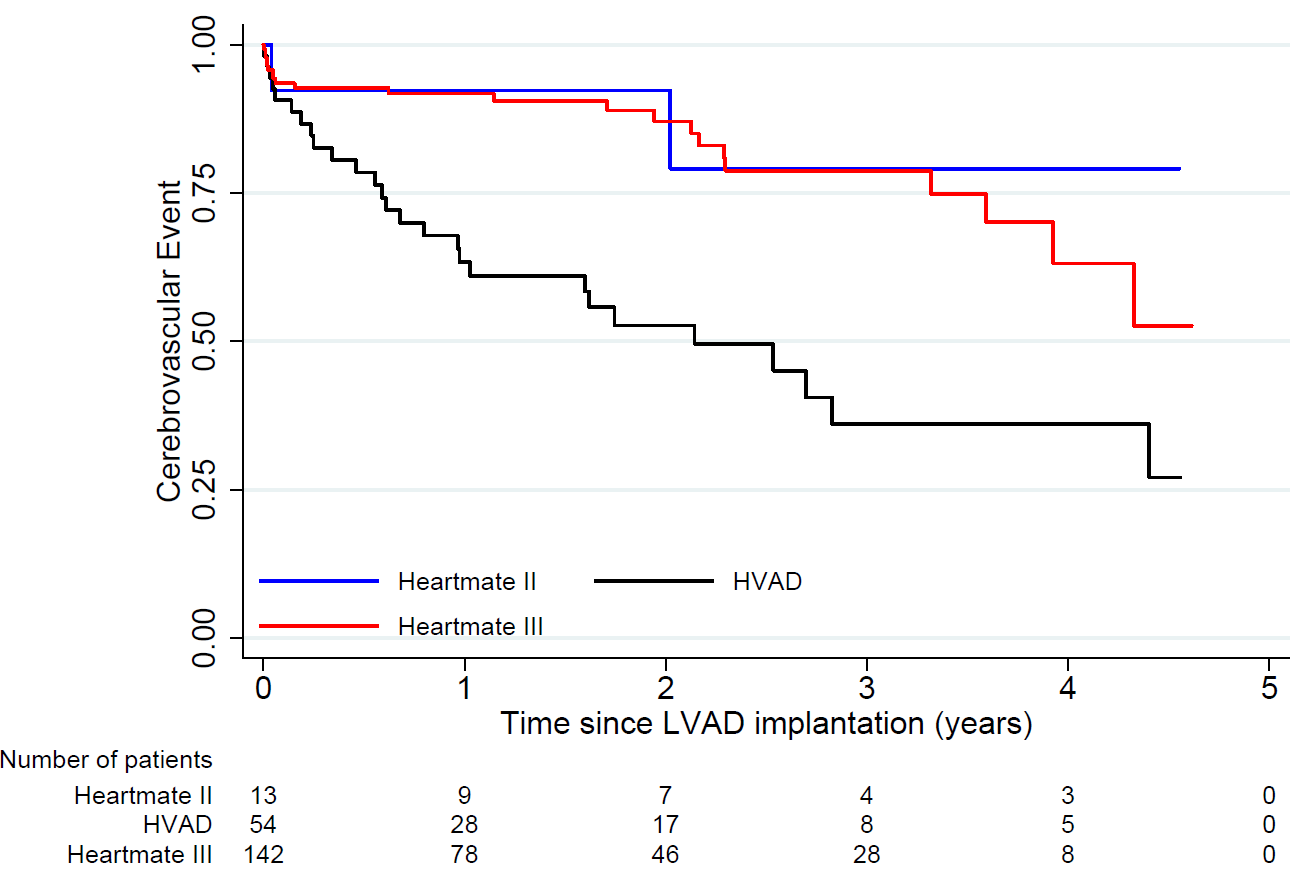
**

**Supplementary Figure 4 – Incidence of Major Adverse Events**

**
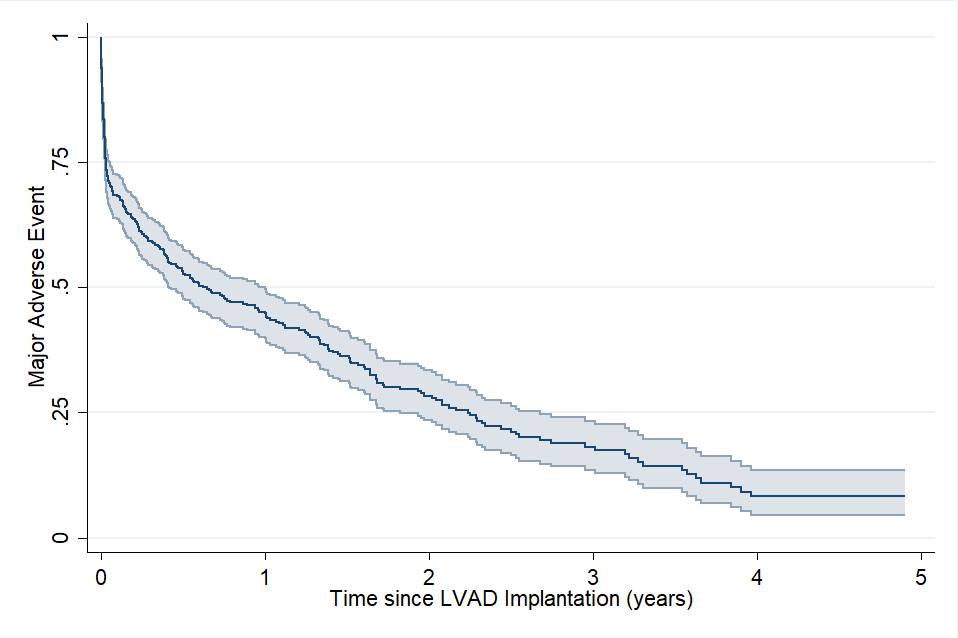
**

Major Adverse events: first occurence of either Major Bleeding, Major Infection, Cerebrovascular event and Device Dysfunction
