# Supplementary material for: Left ventricular assist device implantation and clinical outcomes in the Netherlands
Source: Neth Heart J. 2023 Feb 1;31(5):189–95. doi: 10.1007/s12471-023-01760-9 (PMC10140239; doi:10.1007/s12471-023-01760-9)
Supplement: Supplementary file 2 — Table S1. Major Adverse Events on LVAD support stratified by type of device [file 12471_2023_1760_MOESM2_ESM.docx]

**Table S1. Major Adverse Events on LVAD support stratified by type of device**

|  | **Heartmate II** | **HVAD** | **HeartMate 3** |
| --- | --- | --- | --- |
| **Major Adverse Event, n (per patient year at Risk)**  Device Dysfunction / LVAD Pump Thrombosis  Major Bleeding  Major Infection  Cerebrovascular Events | 31 (0.44)#  10 (0.16)  33 (0.42)  1 (0.02) | 63 (0.21)  140 (0.61)&  261 (0.96)&  17 (0.06)& | 49 (0.08)*&  119 (0.23)*  348 (0.67)*&  5 (0.01)* |

* P < 0.001 vs HVAD

& P < 0.001 vs Heartmate II

# P <0.05 vs HVAD
